# CD59 Underlines the Antiatherosclerotic Effects of C-Phycocyanin on Mice

**DOI:** 10.1155/2013/729413

**Published:** 2013-11-12

**Authors:** Bing Li, Xian-Ming Chu, Ying-Jie Xu, Fan Yang, Cong-Yi Lv, Shu-min Nie

**Affiliations:** ^1^Department of Biology, Medical College of Qingdao University, 38 Dengzhou Road, Qingdao 266021, China; ^2^Department of Cardiology, Affiliated Hospital of Medical College of Qingdao University, Qingdao 266100, China; ^3^Department of Neurological Function Inspection, Affiliated Hospital of Medical College of Qingdao University, Qingdao 266100, China

## Abstract

The effects of C-phycocyanin (C-PC) on atherosclerosis and the regulatory effects of CD59 gene on anti-atherosclerotic roles of C-PC were investigated. Apolipoprotein E knockout (ApoE(−/−)) mice were randomly divided into four groups: control group, C-PC treatment group, CD59 transfection group and C-PC+CD59 synergy group. The mice were fed with high-fat-diet and treated with drug intervention at the same time. Results showed the atherosclerotic mouse model was successfully established. CD59 was over-expressed in blood and tissue cells. Single CD59 or C-PC could reduce blood lipid levels and promote the expression of anti-apoptotic Bcl-2 but inhibit pro-apoptotic Fas proteins in endothelial cells. The expression levels of cell cycle protein D1 (Cyclin D1) and mRNA levels of cyclin dependent protein kinase 4 (CDK4) in smooth muscle cells were restrained by CD59 and C-PC. CD59 or C-PC alone could inhibit the formation of atherosclerotic plaque by suppressing MMP-2 protein expression. In addition, C-PC could promote CD59 expression. So both CD59 and C-PC could inhibit the progress of atherosclerosis, and the anti-atherosclerotic effects of C-PC might be fulfilled by promoting CD59 expression, preventing smooth muscle cell proliferation and the apoptosis of endothelial cells, reducing blood fat levels, and at last inhibiting the development of atherosclerosis.

## 1. Introduction

Atherosclerosis (AS) is a chronic inflammatory and immunologic disease. Immune and nonimmune factors can induce endothelial dysfunction and initiate the occurrence and development of atherosclerosis [1]. Although there have been significant progresses and achievements in the past decade on atherosclerosis, the cellular and molecular pathogenesis of atherosclerosis is still not fully understood. The potential atherogenic role of the complement system, a main effector arm of immunity and inflammation, remains to be determined. It has been accepted that hyperlipemia, hypertension, smoking, secondary hyperlipidemia, genetic factor, and other factors can lead to AS [2]. Currently, seeking for novel antiatherogenic drugs with high efficacy and low toxicity has become the hotspot and challenge in the research field. 

Complement system is involved in occurrence and development of atherosclerosis [3, 4]. In human being, the deposition of membrane attack complex (MAC) was positively correlated with the progress degree of atherosclerotic change. Evidence from human and animal studies indicates that CD59 is relevant in protecting red blood cells from MAC formation and MAC induced phenomena [5]. Others have shown that the MAC also induces the release of monocyte chemotactic protein-114 and activates signaling pathways that promote proliferation of vascular smooth muscle cells [6]. Extensive clinical data showing that MAC colocalized with other complement activation products and immunoglobulins in human atheromas support the notion that MAC may play a pathogenic role in human atherosclerosis [7]. Recently, Yun et al. demonstrated that the deficiency of CD59 sensitizes LDLR−/− mice to develop atherosclerosis [8]. CD59 is considered to play relevant role in restricting MAC formation in mice [9, 10]. CD59 is expressed at the lower level in hematopoietic cells and testes [11, 12] and it has anti-MAC activity in the mouse, especially in the CD59-deficient condition [9, 12–14]. Complement regulatory protein CD59 is a key regulatory factor of MAC, so CD59 may influence the development of AS by inhibiting the assembling of MAC. The underlying mechanism by which CD59 plays a protective role in the pathogenesis of atherosclerosis remains unclear.

C-Phycocyanin (C-PC), a water soluble fluorescent protein pigment [15], is one of the major constitutes of blue-green algae *Spirulina platensis*. C-PC consists of *α* and *β* subunits and the natural existing form is trimeric aggregation (*αβ*)_3_. C-phycocyanin has characteristic absorption peaks in the visible light region 620 nm. The purity of C-phycocyanin is determined by A620 nm/A280 nm and C-PC has practical application value when the ratio is greater than 4 [16]. Some characteristics of C-PC make it well suitable for fluorescence analyses in flow cytometry, histochemistry, immunoassay, and detection of reactive oxygen species [17]. Taking advantage of the characteristics of high water solubility, high contents, and easy extraction, C-PC can be used as natural dyes and is widely applied to cosmetics and food additives [18]. In addition to its widely accepted usages as fluorescent agent in laboratory works and dye in cosmetics and food industries, C-phycocyanin has other medical values including antioxidation [19], antimutagen [18, 20], antivirus [20], antiallergy [21], hepatoprotection [22], antitumor [23, 24], anti-inflammation [25], and immunity boosting effects [23, 26]. Moreover, C-phycocyanin can improve blood lipid metabolism, scavenge free radical [27], and lower blood lipid [28], thus effectively suppressing the occurrence of hyperlipidemia. We speculate that C-phycocyanin might play a potential role in the therapy of atherosclerosis.

In the present study, the effects of C-PC and CD59 on atherosclerotic mice were investigated, and the antiatherosclerotic mechanism of C-PC and regulatory effects of CD59 were discussed.

## 2. Materials and Methods

All studies were approved by the Qingdao University Institutional Animal Care and Use Committee. Animals were housed under standard conditions with ad libitum food and water and a 12 : 12 light : dark cycle at the Qingdao University facilities.

### 2.1. Materials and Regents

 ApoE(−/−) mice (half male and female) were purchased from Beijing Weitong Lihua Experimental Animal Technical Co., Ltd. and *Spirulina platensis* tablets were purchased from Ocean University of China. CD59-pIRES plasmid was provided by Department of Immunology, Medical College of Qingdao University. Liposome Lipofectamine 2000 was purchased from Invitrogen Co. Cholesterols from Sigma; pig bile salts were from Solarbio Co.; rabbit anti-mouse CD59/cyclinD monoclonal antibody was from Santa; EndoFree Plasmid ezFlow Maxiprep Kit was from BIOMIGA Co.; RNAiso PLUS Kit and PrimeScript RT-PCR Kit were from Takara Co. Cdk 4 *in situ* hybridization kit; *in situ *cell apoptosis detection kit were purchased from Boster Biological Engineering Co., Ltd.

### 2.2. Extraction and Purification of C-Phycocyanin from *Spirulina platensis*

*Spirulina spirulina* tablets were fully grounded to powder and soaked in 10 mM PBS for 24 h and churned up at 7,000 ×g at 4°C for 30 min. With repeated freezing at −20°C and thawing at 38°C in the presence of PBS for 6 times, the powder was centrifuged at 7,000 ×g at 4°C for 50 min. The supernatant was precipitated overnight with 50% saturated (NH_4_)_2_SO_4_ solution at 4°C. After centrifugation at 7,000 ×g at 4°C for 1 h, the precipitate was dissolved in and dialyzed against 10 mM PBS. The dialyzate was used as crude C-PC preparation. Then the crude C-PC extracts were purified with HA column chromatography, followed by sephacrylHR-200 gel chromatography and one more time HA column chromatography [16].

### 2.3. Construction of Animal Models and Grouping

Forty ApoE(−/−) mice were fed with high-fat diet consisting of 80% common feed, 15% lard, 2% cholesterols, 1.5% whole milk powders, 1.3% yolk powders, and 0.2% pig bile salts. The mice were randomly divided into 4 groups (half male and female) and all mice were fed with high-fat diet for 12 weeks. Group 1 (control group): mice were fed with high-fat diet. Group 2 (C-PC treatment group): mice were treated with gavage administration of C-PC (100 mg/kg) twice a week for 12 weeks. Group 3 (CD59 transfection group): liposome-coated recombinant CD59-pIRES plasmid (97.5 *μ*g/mL, 0.5 mL/mice) was injected into mice via tail veins, twice a week for 12 weeks. Group 4 (CD59+C-PC synergy group): mice were treated with gavage administration of C-PC suspension together with the tail vein injection of liposome-coated CD59-pIRES plasmid twice a week for 12 weeks. CD59-pIRES plasmid was extracted from E. coli which has been constructed successfully by immunology department [29].

### 2.4. RT-PCR

At the end of the experiment, after fasting for 12 hours, all the mice were anaesthetized and blood was obtained by ocular puncture using micropipette. Total RNA was extracted from whole blood cells using RNAiso PLUS reagent kits and reverse-transcribed by PrimeScript RT-PCR Kit. Then the products were amplified by PCR. Primers were designed based on sequences of human CD59 and GAPDH. The upstream primer of CD59 was 5′-TGGACAATCACAATGGGAATC-3′ and the downstream primer was 5′-TGCTGCCAGAAATGGAGTCAC-3′. The forward primer of GAPDH was 5′-CGTGGAAGGACTCATGACCA-3′, and the reverse primer was 5′-TCCAGGGGTCTTACTCCTTG-3′. PCR reactions were carried out as follows: predegeneration at 94°C for 2 min, 40 cycles of degeneration at 94°C for 30 s, annealing at 47°C for 1 min, and elongation at 68°C for 2 min. Then final elongation was at 68°C for 7 min. PCR products were quantified using Tanon Image Software. CD59 levels were normalized with respect to GAPDH levels.

### 2.5. Western Blot

Twelve weeks later, the tissues including liver, spleen, heart, and aorta of the mice were taken out and rinsed with precooling normal saline. The tissues were mortared in ice-cold lyses buffer. Proteins were collected with centrifugation and the protein concentrations were determined. Proteins were separated by sodium dodecyl sulfate polyacrylamide gel electrophoresis (SDS-PAGE) and then were transferred onto polyvinylidene fluoride (PVDF) membrane. Western blot analysis was carried out by using blocking buffer, rabbit anti-mouse CD59/*β*-actin primary antibody (1 : 400), and HRP-conjugated goat anti-rabbit secondary antibody (1 : 5000), respectively. The chemiluminescence method was used for color development. The products were quantified and analyzed by Image J software.

### 2.6. Biochemical Analysis of Blood Lipid Levels

Serum levels of total cholesterol (TC), triglyceride (TG), apolipoprotein B (ApoB), low-density lipoprotein (LDL), and high-density lipoprotein (HDL) were examined using biochemical methods.

### 2.7. Preparation of Histological Sections

The aortic roots were dissected out and fixed in 4% paraformaldehyde. Serial paraffin sections were made and stained by HE. The formation degree of atherosclerotic plaques was observed under light microscope.

### 2.8. TUNEL Assay

The paraffin slices were conventionally dewaxed and treated with 30% H_2_O_2_ : methanol (1 : 50) at room temperature for 10 min to block the activities of endogenous peroxidase. After washing with distilled water, the slices were digested with proteinase (1 : 200). DNA breaks were labeled sequentially by TdT and DIG-labeled deoxyuridine triphosphate and then were incubated in blocking buffer, biotinylated antidigoxin IgG, streptavidin–biotin complex (SABC), and diaminobenzidine (DAB, for color development) in turns. The apoptotic ratios were counted.

### 2.9. *In Situ* Hybridization

A 32 bp of oligonucleotide probe for CDK4 mRNA of mice (5′-CTGGAGGCCTTTGAACATCCCAATGTTGTACG-3′, labeled with digoxin at 5′ end) was synthesized. The paraffin slices were conventionally dewaxed and treated with 30% H_2_O_2_ : methanol (1 : 50) at room temperature (RT) for 10 min. The CDK4 mRNA was exposed by the functions of the citric acid and concentrated protease. Prehybridization was performed by adding prehybridizational solution (provided in the CDK4* in situ* hybridization kit) on the slides and incubated in wet box at 38°C for 4 h. Then, hybridization liquid containing 40 ng probe was added on the slides which were then incubated overnight and washed with saline sodium citrate. CDK4 mRNA expression was determined by immunohistochemical staining. In brief, the slides were incubated in blocking buffer followed by incubation with biotinylated mouse antidigoxin IgG, SABC, biotinylated peroxidase, and DAB. The slides were observed under light microscope. CDK4 mRNA levels were determined by integrated optical density (IOD) = density (mean) × area.

### 2.10. Immunofluorescence

SABC-Cy3 represents a new generation of immunofluorescence method. Cy3 is more hydrophilic than traditional fluorescence, and thus it has a lower background. In addition, the sensitivity and stability are better. Cy3 emits fluorescence in 568–574 nm and is bright red. The slices were conventionally dewaxed, immersed into citrate buffer, and heated to boiling. Then the slides were incubated with blocking buffer of goat serum (1 : 10), rabbit anti-mouse cyclin D primary antibodies (1 : 50), biotinylated goat anti-rabbit secondary antibodies (1 : 100), and SABC-Cy3 (1 : 100) in turns. After being washed 4 times with PBS, the slides were observed under fluorescence microscope. Protein expression quantities were determined by IOD.

### 2.11. Immunohistochemistry

The slices were conventionally dewaxed and treated with 30% H_2_O_2_ : methanol (1 : 50) at room temperature for 10 min. After washing with distilled water, antigen was restored by heat. The expressions of proteins (Fas/Bcl-2/MMP-2) were examined by immunohistochemical staining. Briefly, after culturing in blocking buffer (provided in the immunohistochemistry kit), the slides were incubated with rabbit anti-mouse Fas/Bcl-2/MMP-2 primary antibodies, respectively (1 : 100, 1 : 100, 1 : 60), followed by incubation with biotinylated goat anti-rabbit secondary antibodies, SABC and DAB. The results were evaluated by light microscopy. The level of protein expression was expressed as IOD.

### 2.12. Statistical Analyses

Results were expressed as mean ± standard error of mean (SEM). Data were analyzed and processed by SPSS17.0 software. Differences among groups were evaluated by a paired *t*-test and *P* < 0.05 was considered as statistically significant.

## 3. Results

### 3.1. The Purity of C-Phycocyanin

The purity of C-PC was gradually increased by the three steps and finally reached 4.54 (A_620_/A_280_). So the purified C-PC could be used in the following experiments.

### 3.2. Blood Cell CD59 mRNA Expressions Were Upregulated in C-PC Treated and CD59 Transfection Mice

**Figure 1:**
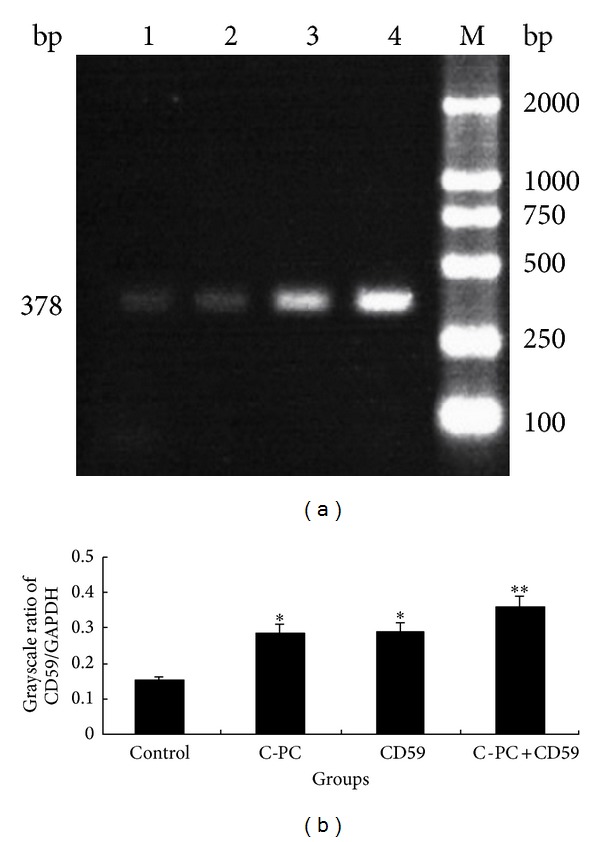
Determination of CD59 mRNA levels in blood cells of four groups of mice. (a) RT-PCR. The amplified CD59 was 378 bp. 1: control group, 2: C-PC treatment group, 3: CD59 transfection group, and 4: C-PC+CD59 synergy group. (b) Grayscale analysis. The expression levels of CD59 mRNA were determined by grayscale ratios of CD59/GAPDH. *P < 0.05 versus control group.

Grayscale ratios of CD59/GAPDH were used to reflect the levels of CD59 mRNA in blood cells of mice. The results showed that the CD59 mRNA levels were different in four groups (Figure 1). CD59 mRNA levels in both C-PC treatment group and CD59 transfection group were significantly higher than that of the control group (*P < 0.05), which suggested that C-PC could promote the expression of CD59 mRNA in blood cells. So, the CD59 mRNA levels in C-PC+CD59 synergy group were the highest among all groups (**P < 0.01).

### 3.3. Tissue CD59 Protein Expressions Were Upregulated in C-PC Treated and CD59 Transfected Mice

**Figure 2:**
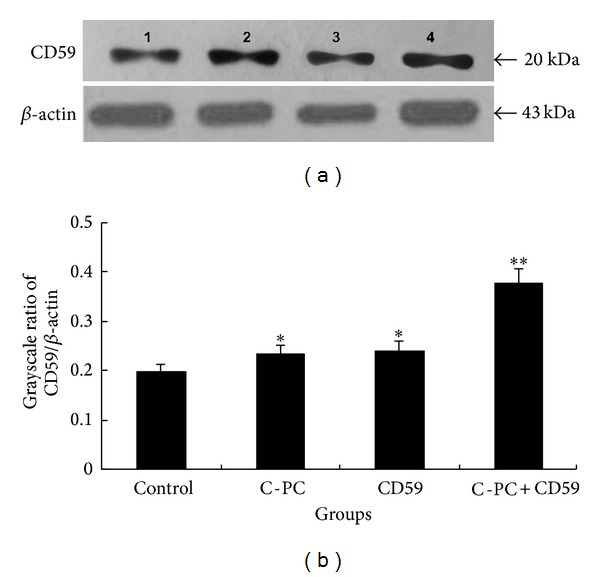
Determination of CD59 protein expression in different groups. (a) Western blot analysis. Two protein bands representing *β*-actin (43 kDa) and CD59 (20 kDa) were observed. Lane 1: control group, Lane 2: CD59 transfection group, Lane 3: C-PC treatment group, and Lane 4: C-PC+CD59 treatment group. (b) Grayscale ratios of CD59/*β*-actin. The grayscale ratios were proportional to the CD59 protein levels. Compared with control group, the grayscale ratios in C-PC treatment group and CD59 transfection group were enhanced. The differences were statistically significant (*P < 0.05). CD59 protein level was further increased in CPC+CD59 synergy group (**P < 0.01). No overt differences were observed between C-PC treatment group and CD59 transfection group.

The expression of CD59 protein was detected by western blot (Figure 2(a)) and the grayscale ratios of CD59/*β*-actin were proportional to the CD59 protein levels. CD59 protein levels were different in four groups. Compared with control group, CD59 protein levels were enhanced in C-PC treatment group and CD59 transfection group, and the differences were significant (*P < 0.05), but the difference between C-PC group and CD59 group was not significant (*P* > 0.05). The CD59 protein level in C-PC+CD59 treatment group was the highest of the four groups (**P < 0.01). The results showed that C-PC could promote the CD59 expression in tissue of mice. The CD59 protein level was elevated after transfection of recombinant CD59-PIRES plasmid, which suggested that CD59 gene had been successfully transfected into the tissue cells of mice (Figure 2(b)).

### 3.4. The Blood Lipids Levels

Compared with normal-diet ApoE(−/−) mice, the levels of TG, TC, Apo B, and LDL were increased in high-fat-diet mice except for HDL, and the differences were significant (*P* < 0.05, Table 1). The results proved that the high-fat-fed mice had a predisposition to hyperlipidemia, which could induce the formation of atherosclerosis. As for ApoB level, there was no significant difference in four experimental groups. Compared with control group, the levels of TG, TC, and LDL in CD59 transfection group and C-PC treatment group were significantly decreased, while HDL level was increased (*P < 0.05). Moreover, the synergy of C-PC and CD59 was more effective than single drug treatment (**P < 0.01, Table 2). C-PC+CD59 could lower the levels of blood lipids, slow down the formation of hyperlipidemia, and finally inhibit the occurrence of atherosclerosis.

### 3.5. Histological Changes in Aorta

**Figure 3:**
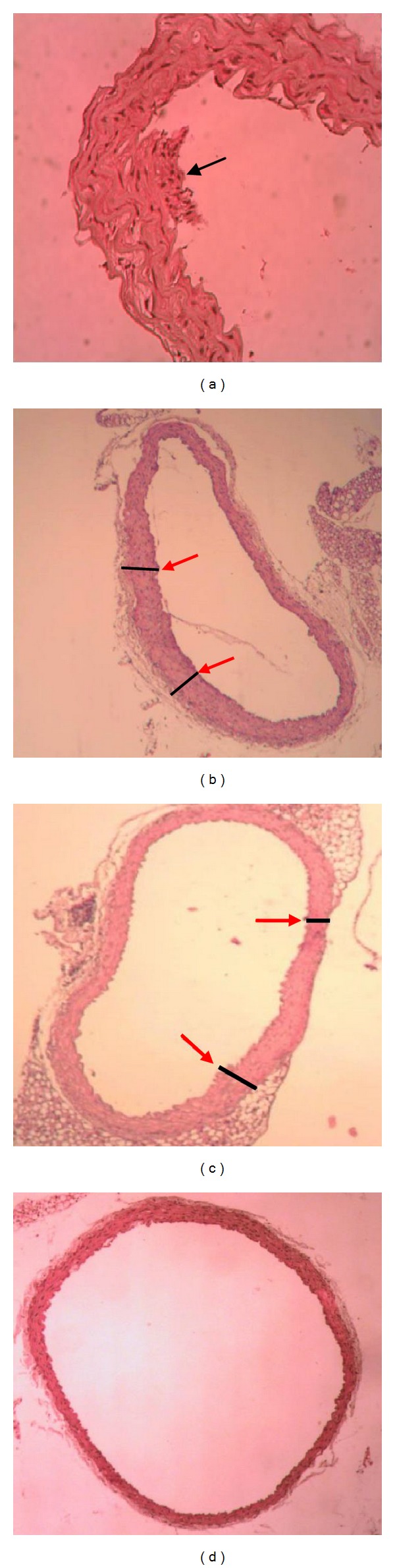
Cross-sections of the aorta root of mice in different groups. The intima was thickened and atherosclerotic plaque built up in high-fat-fed control mice. Compared with the control group, the intima was obviously thinner and the plaque was not found in CD59 transfection group and C-PC treatment group. There was a layer of continuous and smooth endothelial cells on the inside of the blood vessels in CD59+C-PC synergy group with no obvious intima thickening and plaque formation. (a) Control group (×200), (b) C-PC treatment group (×100), (c) CD59 transfection group (×100), and (d) C-PC+CD59 synergy group (×100).

Morphological differences of the aorta specimens in each group were observed under the microscope. The intima was obviously thickened, the integrity was destroyed, and atherosclerotic plaque was built up in high-fat-diet control group. There were more smooth muscle cells found in the intima which showed hyperplasia and transformation. Compared with the control group, the intima was obviously thinner, the integrity was better, and the plaque was not built up in CD59 transfection group and C-PC treatment group (Figures 3(b) and 3(c)). The blood vessel endothelial lining was smooth and continuous in CD59+C-PC synergy group which had no obvious intima thickening and plaque formation (Figure 3(d)). The results showed that C-PC or CD59 could inhibit the buildup of atherosclerotic plaque to some extent, and the inhibitory effects were more significant in CD59+C-PC synergy group, so CD59+C-PC could further slow down and finally inhibit the formation of atherosclerosis.

### 3.6. The Apoptosis Rates Were Lowered in C-PC Treated and CD59 Transfection Mice

**Figure 4:**
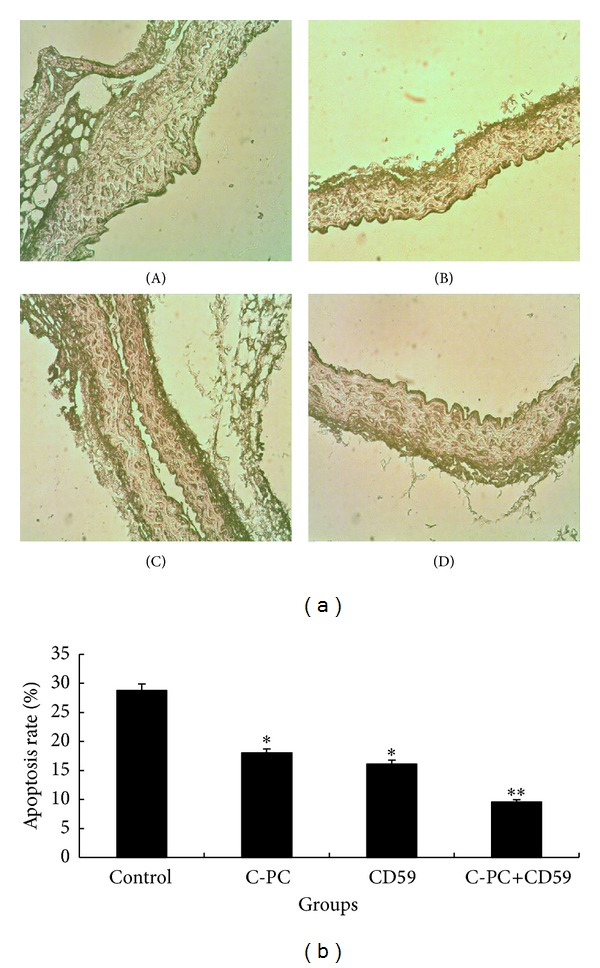
Determination of cell apoptosis in different groups. (a) Photos of TUNEL method (×200). The nuclei of apoptotic cells were dyed brownish yellow. The intima of the aorta in a high-fat-diet control group had brownish-yellow granules. The color was darker in control group than that in C-PC treatment group and CD59 transfection group. However, brownish-yellow granules were the least and the color was the lightest in C-PC+CD59 synergy group. (A) Control group, (B) C-PC treatment group, (C) CD59 transfection group, and (D) C-PC+CD59 synergy group. (b) Comparison of apoptosis rates. Compared with control group, the numbers of apoptotic cells decreased and the apoptosis rates were gradually reduced in single C-PC or CD59 treated group, and the differences were significant (*P < 0.05). But the apoptosis rates were further lowered when the mice were treated with CD59 and C-PC at the same time (**P < 0.01).

Cell apoptosis rates in four groups of mice were detected by TUNEL kit. The positive cells (apoptotic cells) displayed brownish yellow in nucleus. The intima of the aorta in a high-fat-diet control group had brownish-yellow granules. There were more and darker granules in control group than in C-PC treatment group and CD59 transfection group (Figure 4(a)(A–C)). In C-PC+CD59 synergy group, the intima showed the least granules which were more uniformly distributed and showed lightest staining (Figure 4(a)(D)). Results illustrated that compared with control group, the apoptotic ratios in the endothelial cells of aorta were lowered in C-PC treatment group and CD59 transfection group and further decreased in C-PC+CD59 synergy group and apoptotic cells were evenly distributed (Figure 4(b)). Therefore, C-PC or CD59 alone could inhibit the apoptosis of endothelial cells and the inhibitory effects were strengthened in C-PC+CD59 synergy group.

### 3.7. CDK4 mRNA Was Downregulated in C-PC Treated and CD59 Transfected Mice

**Figure 5:**
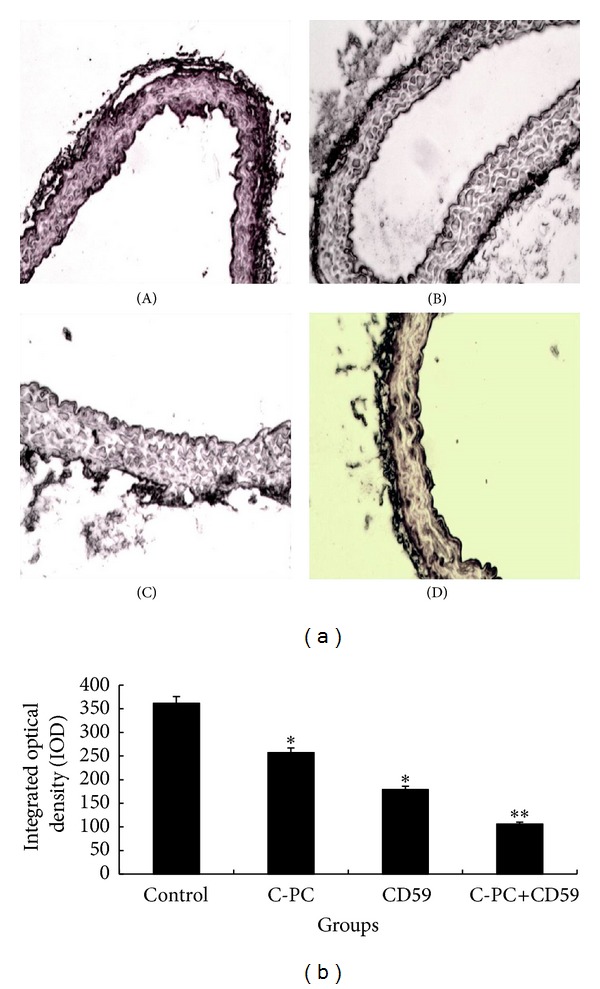
CDK4 mRNA levels in different groups of mice. (a) CDK4 mRNA levels by *in situ* hybridization (×200). The positive cell nucleus was dyed brownish yellow. The color in aortic plaque arose from smooth muscle cells was dark and CDK4 mRNA levels were high in control group. In C-PC treatment group and CD59 transfection group, the nucleus color was lighter and brownish-yellow granules were less than those in control group. Moreover, the color was the lightest and staining intensity was the weakest in C-PC+CD59 synergy group. (A) Control group, (B) C-PC treatment group, (C) CD59 transfection group, and (D) C-PC+CD59 synergy group. (b) IOD values of different groups of mice. *P < 0.05, **P < 0.01 versus control group. IOD values represent the levels of protein expression. Compared with the control group, the IOD values in CD59 or C-PC treated groups were decreased and the differences were significant (*P < 0.05). The IOD values were further enhanced in C-PC and CD59 synergy group and the differences were very significant (**P < 0.01).

 Cdk4 mRNA levels in cells of aorta were detected by *in situ *hybridization. The positive cell nucleus was dyed brownish yellow. In control group, the nucleus color of smooth muscle cells in aortic plaque was dark and CDK4 expression was strong (Figure 5(a)(A)). In C-PC treatment group and CD59 transfection group, the nucleus color of smooth muscle cells was lighter, brownish-yellow granules were less, and distribution was uniform (Figure 5(a)(B,C)). In C-PC+CD59 synergy group, the color was the lightest and staining intensity was the weakest (Figure 5(a)(D)). IOD values represented the CDK4 mRNA levels. Results suggested that C-PC and CD59 could downregulate CDK4 mRNA levels and synergistic inhibitory effects of C-PC+CD59 were significant (Figure 5(b)). 

### 3.8. Cyclin D1 Protein Expressions Were Lowered in C-PC Treated and CD59 Transfection Mice

**Figure 6:**
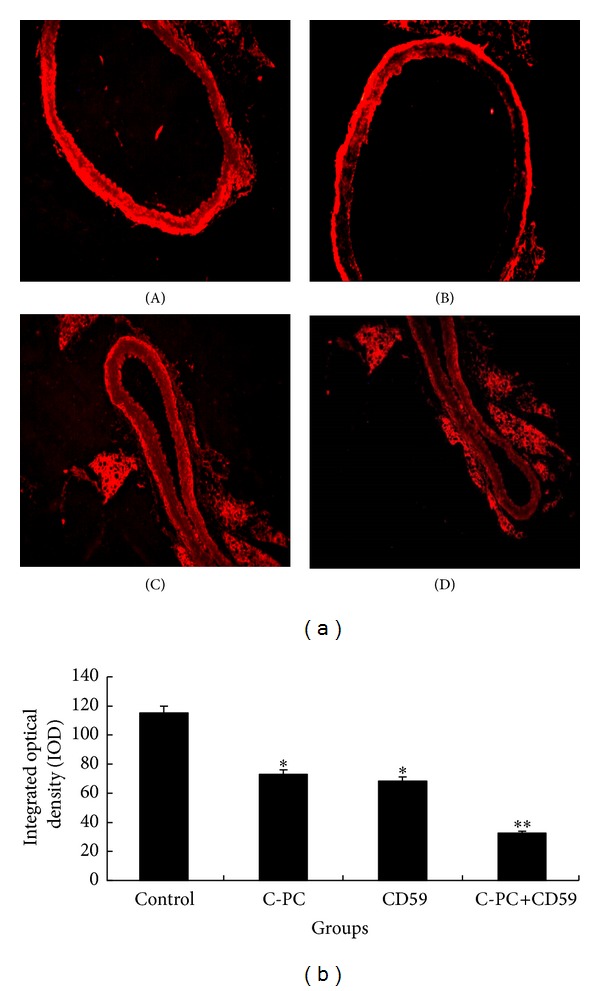
Determination of cyclin D1 by fluoroimmunoassay. (a) The expression of cyclin D1 in four groups of mice (×200). The nuclei of positive cell emitted red fluorescent. Compared with control group, fluorescence intensity in smooth muscle cells of aorta was stronger than that in C-PC treatment group and CD59 transfection group. In C-PC+CD59 synergy group, fluorescence intensity was the weakest and most smooth muscle cells were negative. (A) Control group, (B) C-PC treatment group, (C) CD59 transfection group, and (D) C-PC+CD59 synergy group. (b) Comparison of grayscales in four groups. Grayscales reflect the fluorescence density. Compared with control group, the fluorescence density in C-PC or CD59 treated groups was increased. The differences were statistically significant (*P < 0.05). Moreover, the fluorescence density was further enhanced in C-PC+CD59 synergy group which had significant differences with control group (**P < 0.01).

The expression quantities of cyclin D1 protein in aorta cells were detected by fluorescence immunoassay. The nucleus of positive cell showed red fluorescence. The fluorescence intensity of aortic smooth muscle cells in the control group was stronger than that in C-PC treatment group and CD59 transfection group (Figure 6(a)(A–C)). The fluorescence intensity in C-PC+CD59 synergy group was the weakest and most smooth muscle cells were negatively stained (Figure 6(a)(D)). IOD results suggested that, compared with control group, the cyclin D1 expression levels in the smooth muscle cells of aorta were lowered in C-PC treatment group and CD59 transfection group. The cyclin D1 expression was further decreased in C-PC+CD59 synergy group and even not expressed in certain amount of cells (Figure 6(b)). So single C-PC or CD59 could inhibit cyclin D1 expression levels in smooth muscle cells of aorta and the combined inhibitory effects of C-PC and CD59 were more significant.

### 3.9. The Expression Levels of Proapoptotic Fas Proteins Were Lowered in C-PC Treated and CD59 Transfection Mice

**Figure 7:**
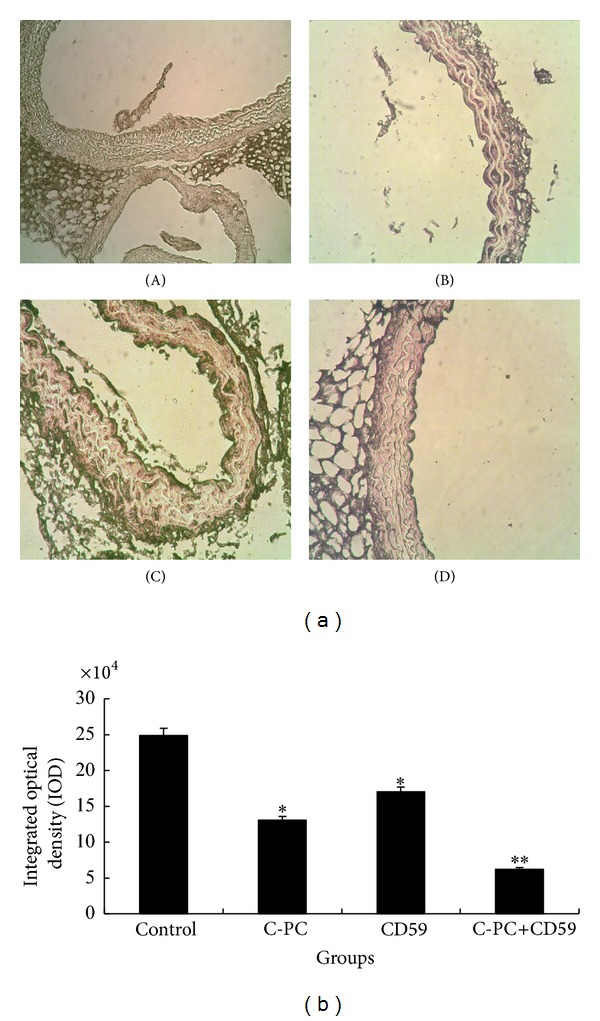
Determination of Fas protein levels. (a) Photos of immunohistochemistry (×200). The cytoplasm of positive cells was dyed brownish yellow. The cellular color in endothelial cells of aorta in high-fat-fed control group was darker than that in C-PC treatment group and CD59 transfection group. While in C-PC+CD59 synergy group, brownish-yellow granules were the least and color was the lightest. (A) Control group, (B) C-PC treatment group, (C) CD59 transfection group, and (D) C-PC+CD59 synergy group. (b) IOD values of different groups of mice. *P < 0.05, **P < 0.01 versus control group. IOD values represent the levels of protein expression. Compared with the control group, the IOD values in CD59 or C-PC treated groups were decreased and the differences were significant (*P < 0.05). The IOD values were further enhanced in C-PC and CD59 synergy group and the differences were very significant (**P < 0.01).

The expression levels of proapoptotic Fas protein in endothelium cells of aorta were detected by immunohistochemistry. The cytoplasm and cell membrane of positive cells were dyed brownish yellow. In high-fat-fed control group, the color of the aortic endothelial cells was darker than that in C-PC treatment group and CD59 transfection group (Figure 7(a)(A–C)). In C-PC+CD59 synergy group, the aortic endothelial cells showed least brownish-yellow granules and color was the lightest (Figure 7(a)(D)). These results suggested that, compared with control group, the expression level of Fas protein in aortic endothelial cells were lowered in C-PC treatment group and CD59 transfection group. Fas protein level was further decreased in C-PC+CD59 synergy group. So C-PC or CD59 alone could lower Fas protein expression in endothelial cells and the inhibiting effects were more obvious in C-PC+CD59 synergy group.

### 3.10. The Expression Levels of Antiapoptotic Bcl-2 Proteins Were Increased in C-PC Treated and CD59 Transfection Mice

**Figure 8:**
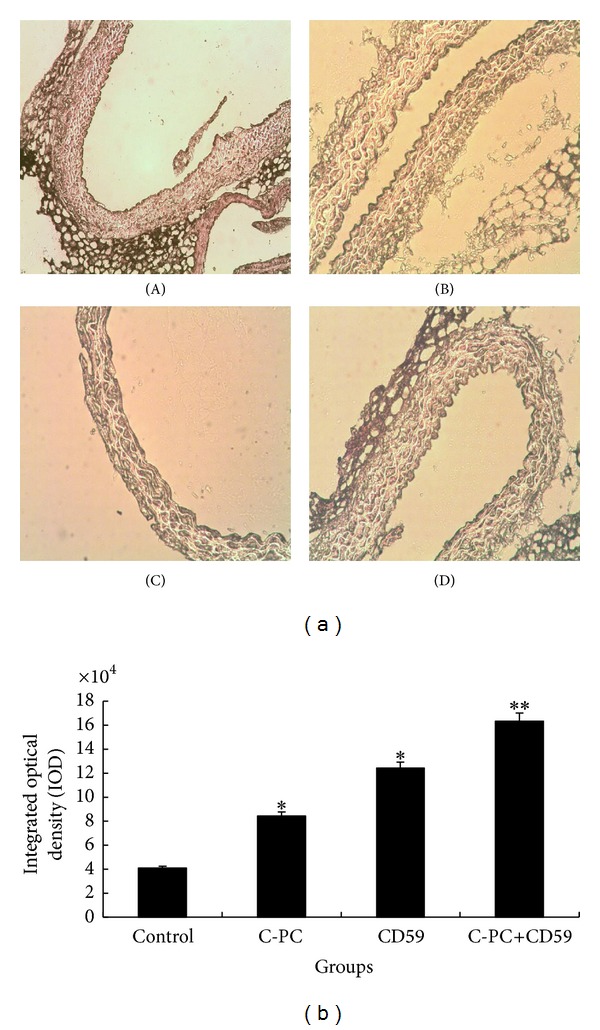
Determination of Bcl-2 protein levels. (a) Photos of immunohistochemistry (×200). In C-PC treatment group and CD59 transfection group, there was no obvious plaque and the stain of endothelial cells was darker than that of control group. In C-PC+CD59 synergy group, brownish-yellow granules were more than other groups and distribution was uniform, and staining was the darkest. (A) Control group, (B) C-PC treatment group, (C) CD59 transfection group, and (D) C-PC+CD59 synergy group. (b) The IOD of Bcl-2. *P < 0.05, **P < 0.01 versus control group. IOD values represent the levels of protein expression. Compared with the control group, the IOD values in CD59 or C-PC treated groups were increased and the differences were significant (*P < 0.05). The IOD values were further enhanced in C-PC and CD59 synergy group and the differences were very significant (**P < 0.01).

The expression levels of antiapoptotic Bcl-2 proteins in endothelial cells of aorta were detected by immunohistochemistry and the cytoplasm of positive cells was dyed brownish yellow. In control group, brownish-yellow particles were observed in intima cells of aorta, but the granule staining was weak. In C-PC treatment group and CD59 transfection group, there was no obvious plaque formation and the staining of endothelial cells was darker than that of control group. In C-PC+CD59 synergy group, the number of brownish-yellow granules was more than those in other groups and the distribution of granules was uniform, and the staining was the darkest (Figure 8(a)). Analysis results of graph were shown in Figure 8(b). The results suggested that, compared with control group, the expression levels of Bcl-2 proteins were higher in C-PC treatment group and CD59 transfection group. Moreover, Bcl-2 protein expression was further upregulated in C-PC+CD59 synergy group.

### 3.11. The MMP-2 Protein Levels Were Lowered in C-PC Treated and CD59 Transfection Mice

**Figure 9:**
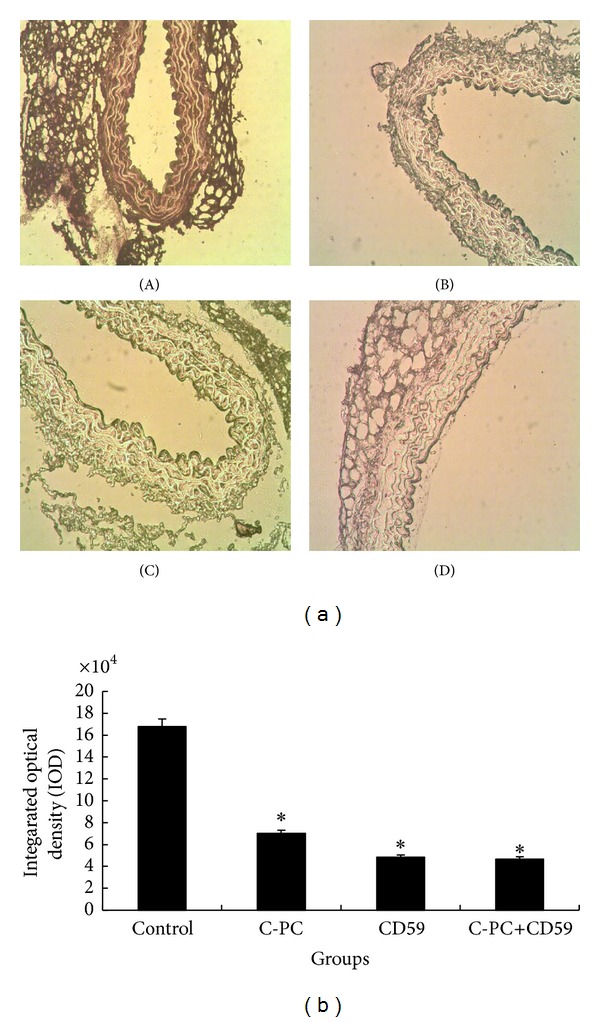
Determination of MMP-2 protein levels. (a) Photos of immunohistochemistry (×200). The membrane and cytoplasm of positive cells showed a brownish-yellow dye after immunohistochemistry. In high-fat-fed control group, plenty of apparent brownish-yellow particles were observed in cells of atherosclerotic plaque and granules were denser than those in other groups. There was no obvious plaque and the stain of endothelial cells in C-PC treatment group and CD59 transfection group was lighter than that of control group. In C-PC+CD59 synergy group, there were no apparent positive cells. (A) Control group, (B) C-PC treatment group, (C) CD59 transfection group, and (D) C-PC+CD59 synergy group. (b) The IOD of MMP-2 protiens. IOD values represent the levels of protein expression. Compared with the control group, the IOD values in CD59 or C-PC treated groups were decreased and the differences were significant (*P < 0.05). The IOD values were further enhanced in C-PC and CD59 synergy group.

The MMP-2 protein expression levels in the endothelium cells of aorta were detected by immunohistochemistry. The cytoplasm of positive cells was dyed brownish yellow. In high-fat-fed control group, plenty of brownish-yellow particles were observed in cells of atherosclerotic plaque and the granules were denser than those in other groups. There was no obvious plaque formation in C-PC treatment group and CD59 transfection group, and the staining of endothelial cells in both groups was lighter than that of control group. There were no obvious positive cells found in C-PC+CD59 synergy group (Figure 9(a)). Quantified analysis results were shown in Figure 9(b). The results suggested that the expression of MMP-2 protein in the atherosclerotic plaque in control group was greater than those in C-PC treatment group and CD59 transfection group. However, MMP-2 protein levels in C-PC+CD59 synergy group were lower than those in other groups.

## 4. Discussions

Atherosclerosis (AS) is a systemic inflammatory disease characterized by the formation of atherosclerotic plaques. Plenty of epidemiological investigations prove that hyperlipidemia is a risk factor for atherosclerosis. Hyperlipidemia is marked by unusual high levels of TC and TG in blood [30]. Atherosclerosis aggravation is caused by the increasing of the plasma cholesterol levels. Now oxidized low-density lipoprotein (ox-LDL) is the most important pathogenic factor for atherosclerosis by damaging the endothelial cells and smooth muscle cells [31]. High-density lipoprotein (HDL) can clear cholesterols away from arterial wall through reverse transfer mechanism, avoid oxidation of LDL by antioxidation, and inhibit the combination of LDL and receptors on endothelial cells to reduce the intake of LDL. So the concentration of LDL is an optimum index to judge atherosclerosis, and the reduction of HDL levels is followed by the aggravation of atherosclerosis. In this study, the concentrations of TG, TC, and LDL in high-fat-diet control group were much higher than those in normal-diet group, but HDL concentration was lower, which indicated the formation of hyperlipemia (Table 1, Figure 4(a)). The intima of aortic root was obviously thickened, the integrity was destroyed, and atherosclerotic plaques formed. All above-mentioned results showed that the atherosclerotic mouse model was successfully established, with formation of atherosclerotic plaques and an increase of serum level in TG, TC, and LDL but not in HDL.

There is positive correlation between deposition of MAC and the development of the lesion. CD59 is a kind of glycosyl phosphatidyl inositol (GPI)-anchoring type glycoprotein. CD59 can combine with C8 and C9 to avoid the formation of MAC C5b-9 complex, and then regulate complement activity to protect host cells from cytolysis caused by complement. CD59 has been discovered to be related with occurrence of trauma, inflammation, and some tumors [32]. Compared with control group, both CD59 mRNA and CD59 protein levels were promoted in CD59 transfection group, which indicated that the recombinant plasmid CD59-pIEPS was successfully transfected into blood cells and tissue cells of ApoE(−/−) mice by liposome embedding and injection into the caudal vein of mice (Figures 1 and 2). Liposome is a ring sealing vesicle composed of lipid double layers and acts as a nonviral gene vector. Liposome has some advantages of non-toxicity, easy-to-use, nonimmunogenicity, and high-transfection efficiency. Target genes were embedded with liposome and injected into vein tail to fulfill some pharmacokinetic effects. Moreover, CD59 transfection could partially reduce blood fat levels, inhibit intimal hyperplasia and buildup of atherosclerotic plaque and further slow down the progress of atherosclerosis (Figure 3). C-PC was also proved to promote CD59 expression, lower blood fat levels, and inhibit intimal hyperplasia and plaque formation. All the above results suggested that CD59 might take part in the antiatherosclerotic effects of C-phycocyanin (C-PC), and the regulatory effects of CD59 gene on anti-atherosclerosis of C-phycocyanin were further discussed.

Cell apoptosis is an initiative cell death under gene regulation and can regulate the balance of cell proliferation in life process [33]. In general, apoptotic cells have obvious morphological changes in nucleus and cytoplasm, including bubbling of cell membrane, degradation of cell nuclei with widespread damage of chromatin, and fragments of DNA caused by Ca^2+^ dependent endonuclease [34]. The degrees of apoptosis were detected by terminal-deoxynucleotidyl transferase mediated nick end labeling (TUNEL) method. The results showed that apoptotic cells can be detected in the intima of all groups of mice and the positive cells were brownish yellow. In high-fat-diet control group, the apoptotic ratios of vascular endothelial cells were the highest and the intima was prone to be destroyed. So medial smooth muscle cells migrated into intima and atherosclerotic plaque formed. The apoptosis rates of endothelial cells in C-PC treatment group and CD59 treatment group were lower than those in control group, so the integrity of aortic intima could be maintained and plaque was difficult to form. The apoptosis rate was the lowest and intima morphology was the most complete in CPC+CD59 synergy group. The results were consistent with the HE staining results of aorta, indicating that both C-PC and CD59 could inhibit apoptosis of endothelial cells and maintain the integrity of the aortic structure. The joint actions of the C-PC and CD59 were more effective to inhibit intimal injury and formation of atherosclerosis plaque. 

The mechanism of apoptosis is very complicated, and studies have shown that Fas and Bcl-2 are the main factors to regulate the apoptosis [35]. Fas has homologous region referred to as “death structure domain” which functions to transduct apoptosis signal, activate a series of cysteine protease, and finally lead to cell death. Studies have found that foam cells in atherosclerotic plaques always have high expression of Fas during the process of cell apoptosis, and apoptosis mediated by Fas can directly induce instability and rupture of atherosclerotic plaques [36]. Bcl-2, located in the inner mitochondrial membrane, endoplasmic reticulum, and nuclear envelope, can block the apoptotic pathway of endothelial cells, leukomonocytes, and neuronal cells. Apoptosis is induced by a series of stimuli. The studies have proved that Bcl-2 plays a very important role in regulating apoptosis and preventing overexpression of apoptosis system [37, 38]. In the present study, the expression quantities of Fas and Bcl-2 proteins were detected in aorta cells by immunohistochemistry. The positive expression levels of Fas in aortic endothelial cells were the highest in high-fat-diet control group which was easy to form atherosclerotic plaque. Fas protein levels were lower in C-PC treatment group and CD59 transfection group and were the lowest in C-PC+CD59 synergy group which was not susceptible to the formation of atherosclerosis. The expression of Bcl-2 gene in vascular intima was weak in control group. Compared with control group, the expression of Bcl-2 gene was higher in C-PC treatment group and CD59 transfection group. Overexpression of Bcl-2 could inhibit apoptosis of endothelial cells and thereby reduce the occurrence of atherosclerosis. The expression of Bcl-2 in C-PC+CD59 was higher than those in C-PC treatment group and CD59 transfection group. The combined effects of C-PC and CD59 could make the turnover and proliferations of aortic cells in a state of homeostasis, and there was no significant apoptosis. 

Cyclin D1 is expressed in G1 phase, which belongs to the highly conservative protein family [39]. CDK4 is an antiapoptotic gene and plays a central role in the regulation of apoptosis. The functional composite cyclin D1/CDK4 can induce cell cycle transition from G1 to S phase, initiate synthesis and replication of DNA, and accelerate cell division. However, aortic medial smooth muscle cells constitute the main part of atherosclerotic plaque, and excessive proliferation of smooth muscle cells is a main reason to induce the formation of atherosclerotic plaque [40]. In the study, CKD4 and cyclin D1 were synthesized in large amounts in smooth muscle cells of control group, and the proliferation of aortic smooth muscle cell sped up. Because of intimal injury, a large number of smooth muscle cells migrated to intima, resulting in atherosclerosis plaque formation. However in C-PC treatment group and CD59 transfection group, CDK4 and cyclin D1 levels were reduced, and cell renewal and proliferation were in balance in CPC+CD59 synergy group. The results demonstrated that C-PC and CD59 can maintain the balance of cell proliferation and apoptosis, hold back the overproliferation of aortic medial smooth muscle cells, and slow down the development of atherosclerotic plaque. Joint inhibitory effects of C-PC and CD59 on the proliferation of smooth endothelial cells were more significant.

Matrix metalloproteinase-2 (MMP-2) is a 66 KDa active ingredient decomposed by 72KDa proenzyme. MMP-2 and its inhibitor TIMP-2 play a very important role in formation and processing on new angiogenesis and has close relationship with the rupture of atherosclerotic vulnerable plaque [41]. MMP-2 is able to degrade the extracellular matrix in atherosclerotic plaque and participates in the formation of atherosclerosis. The expression levels of MMP-2 in the atherosclerotic plaques are significantly increased and affect the stability of plaque, which provides a new method for the screening and diagnosis of atherosclerosis [42]. In our study, the expression quantities of MMP-2 protein in aortic plaque were significantly upregulated in high-fat-diet control group which lead to potential tendency of forming unstable atherosclerotic plaque. But MMP-2 protein levels decreased in C-PC treatment group and CD59 transfection group and were further downregulated in CPC+CD59 synergy group, which was not easy to form the vulnerable plaque. The results were consistent with the aortic morphological results by HE staining (Figure 3). Therefore, only C-PC or CD59 could hold back the expression of MMP-2 gene in a certain extent, thus slowing the formation of atherosclerotic plaque. The effects of combined administration of C-PC and CD59 were more significant.

## 5. Conclusion

In summary, both C-PC and CD59 could reduce blood lipid levels, regulate cell cycle, maintain the stability of the proliferation and apoptosis of aortic cells, and slow down the occurrence and development of atherosclerotic vulnerable plaque. The anti-atherosclerosis mechanism of C-PC was possibly related to its functions of upregulating CD59 expression which inhibit MAC formation, preventing the proliferation of smooth muscle cells and the apoptosis of endothelial cells, decreasing blood lipid levels, and consequently inhibiting the development of atherosclerosis. This study provided a new idea and method for drug and gene therapy of atherosclerosis.

## Figures and Tables

**Table 1:** Comparison of blood lipid levels in normal-diet and high-fat-diet mice.

The levels of blood lipids (mmol/L)	TG	TC	APOB	LDL	HDL
Normal-diet mice	1.29 ± 0.18	2.30 ± 0.25	0.10 ± 0.30	0.27 ± 0.02	3.23 ± 0.25
High-fat-diet mice	2.75 ± 0.10*	20.75 ± 2.05*	0.40 ± 0.03*	4.88 ± 0.76*	2.78 ± 0.05*

*P < 0.05 versus normal-diet mice.

**Table 2:** Comparison of the levels of blood lipids in four groups of mice.

Groups	Indexes (mmol/L)			
	TG	TC	LDL	HDL
Control group	2.75 ± 0.10	20.75 ± 2.05	4.88 ± 0.76	2.78 ± 0.05
C-PC treatment group	1.49 ± 0.10*	11.03 ± 0.13*	3.08 ± 0.16*	3.56 ± 0.19*
CD59 transfection group	1.21 ± 0.05*	12.28 ± 1.08*	3.20 ± 0.12*	3.78 ± 0.08*
CD59+CPC synergy group	0.91 ± 0.04**	3.61 ± 0.04**	0.28 ± 0.03**	4.39 ± 0.04**

*P < 0.05, **P < 0.01 versus control group.

## Abbreviations

C-PC: C-phycocyanin
MAC: Membrane attack complex
ApoE(−/−) mice: Apolipoprotein E knockout mice
CDK4: Cyclin dependent protein kinase 4
AS: Atherosclerosis
HE: Hematoxylin/eosin
GPI: Glycosyl phosphatidyl inositol
TC: Total cholesterol
TG: Triglyceride
LDL: Low-density lipoprotein
HDL: High-density lipoprotein
PVDF: Polyvinylidene fluoride membranes
SABC: Avidin-biotin complex
DAB: Diaminobenzidine
SMC: Smooth muscle cell
IOD: Integrated optical density.
